# Tumor Response to Stroma-Modifying Therapy: Magnetic Resonance Imaging Findings in Early-Phase Clinical Trials of Pegvorhyaluronidase alpha (PEGPH20)

**DOI:** 10.21203/rs.3.rs-3314770/v1

**Published:** 2023-09-05

**Authors:** Andrés M. Arias-Lorza, James R. Costello, Sunil R. Hingorani, Daniel D. Von Hoff, Ronald L. Korn, Natarajan Raghunand

**Affiliations:** Moffitt Cancer Center; Moffitt Cancer Center; University of Nebraska Medical Center; Translational Genomics Research Institute (TGen); Imaging Endpoints Core Lab; Moffitt Cancer Center

**Keywords:** Pegvorhyaluronidase alpha, hyaluronan, cancer, clinical trial, magnetic resonance imaging

## Abstract

Pre-clinical and clinical studies have shown that PEGPH20 depletes intratumoral hyaluronic acid (HA), which is linked to high interstitial fluid pressures and poor distribution of chemotherapies. 29 patients with metastatic advanced solid tumors received quantitative magnetic resonance imaging (qMRI) in 3 prospective clinical trials of PEGPH20, HALO-109–101 (NCT00834704), HALO-109–102 (NCT01170897), and HALO-109–201 (NCT01453153). Apparent Diffusion Coefficient of water (ADC), T1, *k*^*trans*^*, v*_*p*_*, v*_*e*_, and iAUC maps were computed from qMRI acquired at baseline and ≥ 1 time point post-PEGPH20. Tumor ADC and T1 decreased, while iAUC, *k*^*trans*^, *v*_*p*_, and *v*_*e*_ increased, on day 1 post-PEGPH20 relative to baseline values. This is consistent with HA depletion leading to a decrease in tumor water content and an increase in perfusion, permeability, extracellular matrix space, and vascularity. Baseline parameter values that were predictive of pharmacodynamic responses were: ADC > 1.46×10^−3^ mm^2^/s (Balanced Accuracy (BA) = 72%, p < 0.01), T1 > 0.54s (BA = 82%, p < 0.01), *iAUC* < 9.2 mM-s (BA = 76%, p < 0.05), *k*^*trans*^<0.07min^−1^ (BA = 72%, p = 0.2), *v*_*e*_<0.17 (BA = 68%, p < 0.01), and *v*_*p*_<0.02 (BA = 60%, p < 0.01). Further, *v*_*e*_<0.39 at baseline was moderately predictive of response in any parameter (BA = 65.6%, p < 0.01 averaged across patients). These qMRI biomarkers are potentially useful for guiding patient pre-selection and post-treatment follow-up in future clinical studies of PEGPH20 and other tumor stroma-modifying anti-cancer therapies.

## Introduction

The extracellular matrix (ECM) of solid tumors can contain significant amounts of hyaluronan or hyaluronic acid (HA), a linear glycosaminoglycan with repeating disaccharide units of glucuronic acid and N-acetylglucosamine. HA binds up to 15 water molecules per disaccharide, forming a viscoelastic gel-fluid phase that has been mechanistically linked to high interstitial fluid pressure (IFP) in tumors. A dense ECM and high IFP, coupled with dysfunctional tumor microvasculature, leads to poor distribution into the tumor mass of systemically administered therapeutics^1–3^. High tumor levels of HA can also promote intratumoral hypoxia and acidity^4^, further contributing to poorer prognoses. Therefore, targeting HA in the ECM of tumors with high HA accumulation is therapeutically relevant.

Pegvorhyaluronidase alpha (PEGPH20) represents an investigational anticancer therapeutic that was developed to target and degrade tumor HA^5,6^. In vitro and preclinical studies have shown that PEGPH20 depletes HA, leading to decreased IFP, increased perfusion, decreased water, increased micro-vessel area, reduced hypoxia, increased pH, and changes in water diffusion, among other therapeutic events in tumor^2,4,7–9^. Tumor growth inhibition and improved survival^10,11^, and an increase in the intra-tumoral concentration of concomitant chemotherapies^7,9^, following PEGPH20 has been reported in multiple mouse models.

These results led to the clinical development and testing of PEGPH20. Early phase clinical studies showed a decrease in tumor HA and an increase in tumor perfusion resulting in increased survival in patients with high tumor HA receiving PEGPH20 in combination with the chemotherapy drug gemcitabine (GEM)^5,6,12,13^. In a phase 1b study of metastatic pancreatic ductal adenocarcinoma (PDA), stratification of patients for baseline tumor HA content indicated that median progression-free survival (PFS) and overall survival (OS) were longer in patients with high HA tumors than in those with low HA tumors^6^. In a randomized phase II study of PEGPH20 plus nab-paclitaxel/GEM (PAG) vs. nab-paclitaxel/GEM (AG), in patients with high intratumoral HA, the objective response rate was 45% and median overall survival was 11.5 months in patients who received PAG, compared with 31% and 8.5 months in patients who received AG^13^. Based on results from the Phase 1b and Phase II studies, a randomized, double-blind phase III study of PEGPH20 + AG vs. placebo + AG was conducted in patients with high-HA metastatic PDA (HALO-109–301, NCT02715804). Results from 492 patients indicated that the addition of PEGPH20 to AG increased the objective response rate (ORR) but did not improve the duration of response, OS, or PFS^14^. In another phase II study of metastatic PDA, patients with unknown tumor HA status were randomly assigned to receive either the combination of mFOLFIRINOX plus PEGPH20 or mFOLFIRINOX alone (n = 138). The combination with PEGPH20 was less tolerated and increased the likelihood of missed doses^15^. With similar obstacles encountered with other agents targeting tumor stroma in PDA (reviewed in ^16^), targeting desmoplasia alone appears insufficient, and other properties such as lack of significant neoantigens, low tumor mutational burden, and epithelial-to-mesenchymal transition are hypothesized to play a role^17^. Another confounding factor may be that the tumor stroma, though a physical barrier hampering drug delivery, may also have protective effects in restraining tumor growth and progression^15^. Continued clinical development of PEGPH20 has been halted, and a need for additional pre-clinical and retrospective analyses is indicated to improve our understanding of the failures of tumor stroma remodeling as a therapeutic strategy^14^.

A clear need is development of criteria for selecting patients who are likely to benefit from the addition of PEGPH20 to their therapeutic regimen. Tumor HA status on biopsy-based immunohistochemical scoring may be inadequate for prognostication^18^. Biopsy-based assays are invasive and cannot capture intratumoral spatial heterogeneity or inter-lesion heterogeneity in oligometastatic disease^19^ as was the case in HALO-109–301^14^. Radiologic imaging can provide non-invasive longitudinal assessment of tumor heterogeneity over the entire 3D volumes of multiple tumors in a patient. For example, Apparent Diffusion Coefficient (ADC) estimated from Diffusion Weighted (DW)-MRI provides a non-invasive measure of tumor water content and mobility, both properties reported to be affected by PEGPH20^8,20–22^. Tumor perfusion and microvascular permeability (*k*^*trans*^) calculated from Dynamic Contrast-Enhanced (DCE)-MRI has been reported to increase following PEGPH20 in pre-clinical and clinical studies^5,6,9^.

Here we report the first complete analysis of DW-MRI and DCE-MRI collected in three early-phase clinical trials of PEGPH20 (HALO-109–101, HALO-109–102, and HALO-109–201). On a relatively large sample of patients with advanced solid tumors, we computed maps of ADC, T1, *k*^*trans*^, plasma volume fraction (*v*_*p*_), extracellular extravascular volume fraction (*v*_*e*_), and Area-Under-the-Gadolinium-Concentration-Curve-at-90s (iAUC). We report both early post-PEGPH20 prognostic, and pre-PEGPH20 predictive, biomarkers that could be used to select patients who are good candidates for PEGPH20 therapy.

## Results

### VOI analysis

Ten patients from HALO-109–101 and 13 patients from HALO-109–102 have DW-MRI available from multiple scan dates; however, in one patient (HALO-109–102, 102–002-103) ADC could not be computed as b = 0 s/mm^2^ images were not acquired. Thus, ADC of a total of 40 tumors in 22 patients were analyzed, out of which ADC replicates at baseline were available for 18 patients with a total of 28 tumors. At baseline, ADC from patient 101–003-103 was not included as the b values used for DW-MRI in this subject (0 & 1000 s/mm^2^) were a deviation from the prescribed study protocol values (0 & 450 s/mm^2^). Also, ADC at baseline for patient 102–002-113 could not be calculated as the b = 0 s/mm^2^ images were not acquired. Therefore, changes in ADC at baseline from 20 patients with a total of 31 tumors were analyzed. Further, 19 patients with multiple scan dates of T1w-MRI and DCE-MRI were available (13 from HALO-109–102 and 6 from HALO-109–201); however, in one patient from HALO-109–102 (patient 102–002-102) T1 maps could not be obtained as T1w-MRI were acquired using one FA only. Additionally two tumors in patient 201–007-405 were not clearly visible on T1w-MRI. Therefore, T1 from 18 patients with a total of 26 tumors, and DCE parameters (iAUC, *k*^*trans*^, *v*_*p*_, and *v*_*e*_) from 19 patients with a total of 29 tumors, were analyzed. Patient 102–003-104 did not have T1w-MRI images at baseline. Patients 102–003-104 and 102–003-117 were left out of the DCE-MRI analysis at baseline due to missing T1w-MRI and DCE images, respectively. Therefore, changes in T1 in 24 tumors from 17 patients and changes in DCE-MRI parameters in 26 tumors from 17 patients were analyzed. In total, 29 patients were analyzed, and a summary of patient data is described in Table 1. Drug pharmacokinetics and survival information per patient are described in Table 1 Supplementary Material A.

**Figure 1:**
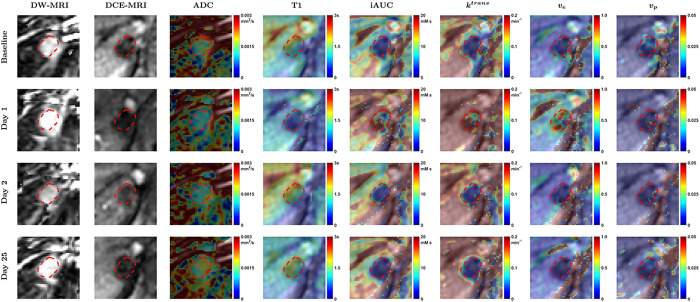
DW-MRI (b = 0 s/mm^2^), DCE-MRI images (2^nd^ time point after AIF peak), and parameter maps per visit of a metastatic colorectal cancer tumor located in the liver of patient 102–003-105 (tumor 2). Contour annotation of tumor is shown as dashed red line. After PEGPH20 (Day 1) a decrease in ADC and T1, and an increase in iAUC, k^trans^, and v_e_, within the tumor are observed. An increase in v_p_ is observed in few pixels.

Figure 1 shows parameter maps per visit of patient 102–003-105 (tumor 2) with a colorectal cancer metastasis in the liver. At Day 1 following PEGPH20, a relatively uniform decrease in ADC and T1, and an increase in iAUC, *k*^*trans*^, and *v*_*e*_, within the tumor are observed. An increase in *v*_*p*_ is observed in a few pixels. Over the following days these parameters tended to return to baseline. Per-patient HTML reports that depict all slices of raw qMRI images, processed qMRI images, and computed maps of ADC, T1, iAUC, *k*^*trans*^, *v*_*p*_, and *v*_*e*_, from all scan dates of each subject, have been publicly shared (Supplementary Material B).

**Figure 2:**
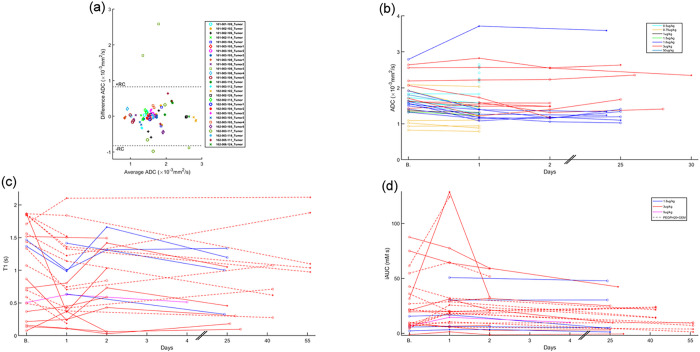
Median parameter changes at each time point for each tumor. In all parameter change figures, the symbol * indicates a true significant change based on the RC. In the x-axes, “B.” indicates the baseline time point. In ADC, Bland-Altman plot describing median ADC repeatability is also shown, where each marker/color represents a baseline ADC replicate difference.

**Figure 3:**
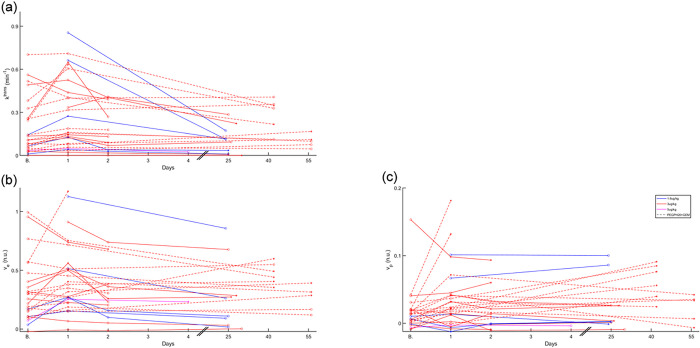
Median parameter changes at each time point for each tumor. In all parameter change figures, the symbol * indicates a true significant change based on the RC. In the x-axes, “B.” indicates the baseline time point.

Median parameter changes in tumor are depicted in Figs. 2 and 3. Changes outside the Repeatability Coefficient (RC) range are also depicted, indicating significant differences with respect to baseline. A Bland-Altman plot of ADC measurement variability described by RC for 28 tumors from 18 patients with available ADC replicates at baseline is shown in Fig. 2a. For the remaining 3 (= 31–28) tumors, we applied the whole data RC = 0.8×10^−3^*mm*^2^/*s*. In the 31 tumors that were analyzable, a decrease in ADC on day 1 relative to baseline was observed in 24 tumors: 8 tumors were below −RC, 2 tumors above + RC, and the remaining did not have a significant change. In the next visit, ADC of 6 out of 16 tumors were below −RC and of 1 tumor was above + RC. In the last visit, ADC of two and one out of 9 tumors were below −RC and above + RC respectively (Fig. 2b).

On day 1 relative to baseline, tumor T1 decreased below –RC in 13 out of 24 tumors, and increased above + RC in 2 tumors. At the next visit, the corresponding numbers were 5 tumors below −RC and 4 tumors above + RC, out of 22 total evaluable tumors. In the last visit, T1 of 1 each of 7 tumors were below −RC and above + RC (Fig. 2c).

On Day 1 relative to baseline, 3 and 10 out of 26 tumors exhibited tumor iAUC changes that were below −RC and above + RC respectively. At the following visits, tumor iAUC of 8 and 3 out of 24 tumors, and 5 and 0 out of 6 tumors, were below −RC and above + RC (Fig. 2d).

Tumor *k*^*trans*^ of 0 and 9 tumors on day 1, 2 and 3 tumors on visit 2, and 4 and 0 tumors in the last visit, were below −RC and above + RC respectively relative to baseline (Fig. 3a).

Tumor *v*_*e*_ of 2 and 10 tumors on day 1, 5 and 6 tumors on visit 2, and 2 and 0 tumors in the last visit, were below −RC and above + RC, respectively relative to baseline (Fig. 3b).

Tumor *v*_*p*_ of 4 and 12 tumors on day 1, 7 and 11 tumors on visit 2, and 1 tumor each at the last visit, were below −RC and above + RC respectively relative to baseline (Fig. 3c).

The results in Figs. 1–3 suggest a response to PEGPH20 treatment that manifests as a decrease in ADC and T1 on day 1, possibly from a decrease in tumor water content due to HA depletion^20^. Results also show an increase in iAUC, *k*^*trans*^, and *v*_*p*_ on day 1, suggesting an increase in perfusion, permeability, and vascularity. *v*_*e*_ also increased after treatment, suggesting a release of ECM space.

**Figure 4:**
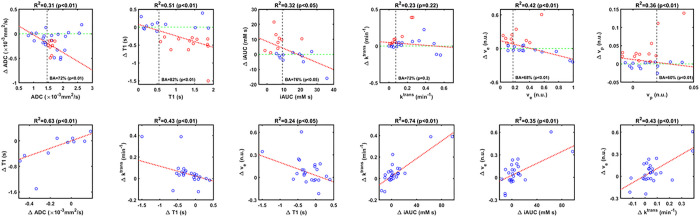
Scatter plots of median parameter changes (Day 1 – Baseline) to parameter at baseline. Red circles indicate significant changes below −RC for ADC and T1, and above +RC otherwise. A linear regression is shown in red dotted line (its R^2^ and p-value (F-test) are above each plot). The vertical dotted black line indicates the threshold that maximizes BA for significant changes at day 1. BA and its p-value (McNemar test) are also shown. Bottom row shows scatter plots between median parameter changes (Day 1 – Baseline) that are significantly correlated. Some outliers are not shown to improve visualization.

Correlations of median changes between parameters are shown in Fig. 4 (bottom row); whereas changes in ADC and T1 are significantly correlated to one another, changes in *v*_*e*_ are correlated with changes in most other parameters.

Scatter plots of median changes vs. parameters at baseline are shown in Fig. 4 (top row). Most points in Δ*ADC* and Δ*T*1 are negative, confirming the tendency of ADC and T1 to decrease following PEGPH20 treatment. Also, there is a negative correlation, which suggests a dependency of response to baseline values. Significant changes in ADC and T1 below −RC are more likely in tumors with baseline ADC values above 1.46 × 10^−3^*mm*^2^/*s* and baseline T1 values above 0.54 s. Balanced Accuracy using these thresholds for both ADC and T1 are 72% and 82%.

Most changes in the other parameters are positive on day 1 (Fig. 4). For iAUC, there is a negative correlation to baseline values with most significant changes above + RC happening in tumors with baseline values below 9.2 *mM-s* (BA = 76%). Changes in *v*_*e*_ and *v*_*p*_ are also negatively correlated to baseline, with thresholds to separate significant and non-significant changes above + RC at 0.17 (BA = 68%) and 0.02 (BA = 60%) respectively. Changes in *k*^*trans*^ were non-significantly negatively correlated to baseline values, with an optimal threshold of 0.07 min^−1^ (BA = 72%, *p >* 0.05).

No clear relation was observed between changes in quantitative MRI parameters and drug dose or drug pharmacokinetics (Cmax, Cmin, AUC). Also, no relation was observed with HA levels in stained tumors and survival (Tables 2–4 in Supplementary Material A).

**Figure 5:**
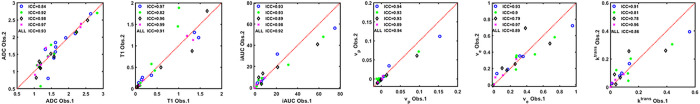
Inter-observer variability analysis. Each point represents a median parameter for each tumor annotated by observer 1 (NR) and observer 2 (JRC) on a subset of 5 patients (102–003-105, 102–003-118, 102–006-107, 102–006-110, 102–006-124). Blue circles are the baseline values, green * are the day 1 values, black diamonds are the visit 2 values, and purple × are the last visit. Intra-class correlations (ICC) for each visit and all points are depicted. Units are ×10^−3^ mm^2^/2 for ADC, s for T1, mM-s for iAUC, min^−1^ for k^trans^, while both v_e_ and v_p_ are dimensionless.

To investigate the robustness of the results with respect to the manual annotations, an expert radiologist (JRC) re-annotated tumors in a subset of five patients on DW-MRI and DCE-MRI at each visit. Correlations of median tumor parameters between observers are shown in Fig. 5, revealing very high correlations (*ICC* ~ 0.9).

### Pixel-wise analysis

A pixel-wise analysis of all parameter values was carried out to develop a multivariable model for predicting post-PEGPH20 response in any parameter from baseline parameter values. Eleven patients have lesions imaged using DWI, T1, and DCE-MRI; however, in three patients either ADC, T1 or DCE parameters could not be computed at baseline; and in two patients (102–002-115, 102–002-126) the sequences were not all acquired in the same view. In all, it was possible to co-register DWI, T1, and DCE-MRI sequences across scan dates in six patients, as shown for one patient in Fig. 1, and these were used in the pixel-wise analysis. Parameter changes in pixels from these six patients between Day 1 and baseline were visualized using Principal Component Analysis (PCA) to identify two clusters (Fig. 2 in Supplementary Material A). As in the VOI analysis, each tumor pixel was considered to either be a pharmacodynamic responder (p-responder) or non-responder (p-non-responder) based on its parameter changes relative to its RC.

For ADC we applied the pixelwise RC per tumor when baseline repeats were available, or otherwise the group pixel-wise RC = 1.4 × 10^−3^*mm*^2^*/s* obtained from all patients using equal numbers of pixels per patient. For the other parameters we used the same RC as in the VOI analysis. A pixel with a parameter change between Day 1 and baseline that is below −RC for ADC and T1, or above + RC for the other parameters, was labeled a “p-responder” pixel, and otherwise a “p-non-responder” pixel.

Using the pixelwise baseline parameter values as independent variables, and the pixel class (p-responder or p-non-responder) as the dependent variable, we trained a decision tree model using the machine learning software Weka (www.cms.waikato.ac.nz/ml/weka/). To mitigate model over-fitting, we separated pixels into two sets, one for training and one for validation. To avoid biasing results towards larger tumors with more pixels, during the training process we sampled an equal number of pixels per patient, equivalent to 85% pixels from the smallest tumor, with the remaining pixels used for validation. Additionally, to avoid problems with unbalanced data, the sampling process during model training was randomized to select equal numbers of “p-responder” and “p-non-responder” in the training set. After each training iteration, the resulting decision tree was applied to the validation set, and a BA per patient was obtained, and the process was repeated multiple times with random sampling from the six patients. After this process, the optimal model was a simple rule: pixels with baseline *v*_*e*_ < 0.39 are predicted to be “p-responder”, and otherwise predicted to be “p-non-responder” (average BA = 74.4% in the validation set, average training BA = 69.2%). As each patient contributed data to the training and validation sets, we performed a leave-one-patient-out cross-validation to evaluate this simple model, obtaining an evaluation average BA = 65.6% across patients (Table 5 in Supplementary Material A).

## Discussion

Pre-clinical studies proved that PEGPH20 successfully depletes intratumoral HA^2,4,7–9,11^, resulting in higher concentrations of concomitant chemotherapies in experimental tumors^7,9^. These results led to clinical studies where, despite early success in phase I/II studies showing survival benefits^6,12,13^, follow-up phase II/III studies failed to show improved outcomes for patients treated with PEGPH20 vs. placebo^14,15^. Reasons for these failures include either failure to pre-select patients^15^, or pre-selection of patients with oligometastatic disease using the HA status of only a single tumor^14^. These failures also underscore the need for improved understanding of tumor stroma remodeling following PEGPH20 treatment^14^. In this work we have sought to address both these needs by investigating post-PEGPH20 changes in the tumor microenvironment using qMRI, and identifying qMRI parameters for informing patient pre-selection. First, we analyzed changes of several tumor properties measured by qMRI, namely, ADC, T1, iAUC, *k*^*trans*^, *v*_*e*_, and *v*_*p*_, from patients enrolled in three early-phase clinical studies of PEGPH20. Second, we present a pharmacodynamic response predictor that could potentially be useful as a MRI biomarker to pre-select patients who are good candidates for combination chemotherapy with PEGPH20.

Tumors that were treated with PEGPH20 exhibited an acute decrease in ADC and T1, and these decreases were correlated with each other. This finding is consistent with pre-clinical reports of post-PEGPH20 decrease of ADC in breast tumors^20,22^ and decrease of water content in pancreatic tumors^2^. A post-PEGPH20 decrease in ADC was also reported in a preliminary analysis of clinical data^21^. A decrease in ADC and T1 suggests a decrease in water due to HA depletion after PEGPH20, as shown by DuFort et al.^2^. A post-PEGPH20 increase in iAUC, *k*^*trans*^, *v*_*e*_, and *v*_*p*_ was also observed in tumors in our study. Post-PEGPH20 increase in tumor *k*^*trans*^ has previously been reported in pre-clinical studies^9^ and in a clinical study of a limited subset of patients^5,6^. A decrease in HA could release tumor interstitial pressure, resulting in increased vascular space causing a higher *v*_*p*_, resulting in a higher blood flow manifesting as increased iAUC and *k*^*trans*^. Depletion of HA would release ECM space, which would manifest as increase in *v*_*e*_. We observed most parameter changes to be significantly correlated to changes in *v*_*e*_, indicating that a change in *v*_*e*_ is the most relevant early response biomarker and tumor property that triggers other changes such as the increase in tumor perfusion and permeability, something that was also confirmed in the pixel-wise analysis where *v*_*e*_ at baseline was predictive of response in any parameter.

A change in any MRI-measured parameter was considered significant if the median change was outside the RC, a measure of parameter variability in repeated scans. For ADC, in most cases an RC per tumor was obtained from repeated scans acquired at baseline. For other parameters, we used literature RC values since repeated T1w-MRI and DCE-MRI scans were not available. A decrease of ADC and T1 below −RC, and an increase in iAUC, *k*^*trans*^, *v*_*e*_, and *v*_*p*_ above + RC, at Day1 post-PEGPH20 relative to baseline values, were considered a pharmacodynamic response to treatment with PEGPH20. This approach could be used at follow-up to determine if a patient is responding to PEGPH20.

We did not observe correlations between parameter changes and either changes in HA or survival, though such analyses were limited by only a few biopsied tumors and good responders by RECIST criteria that were available in our data set. qMRI parameter changes also did not correlate with PEGPH20 dose, which is unsurprising since PEGPH20 is an enzyme and therefore dose-response would not be characterized by the Law of Mass Action.

We identified threshold values of each parameter at baseline for predicting pharmacodynamic response. Baseline parameter values that maximized sensitivity and specificity for prediction of response to PEGPH20 were: *ADC >* 1.46 × 10^−3^mm^2^/s, *T*1 *>* 0.54 s, *iAUC <* 9.2 *mM s, k*^*trans*^*<* 0.07 *min*^−1^, *v*_*e*_*<* 0.17, and *v*_*p*_*<* 0.02. These relations produce significant accuracies above 70% in most cases. High ADC would be expected in tumor regions with high water content^23^ such as would be associated with tumor HA. High tumor water content would also be associated with longer tumor T1 values. Low iAUC and *k*^*trans*^ suggest low perfusion and permeability, while low *v*_*p*_ indicates low vascularity such as might be the case in tumor microenvironments with microvascular compression due to high interstitial pressures stemming from high HA. A low *v*_*e*_ indicates a small ECM space likely due to high HA. Baseline values of these parameters can be used to pre-select patients with tumors that are likely to respond to PEGPH20, and would therefore experience improved tumor penetration by intravenously administered small molecule chemotherapeutics upon pre-treatment or co-treatment with PEGPH20. In this context, our results indicate that tumor pixels with *v*_*e*_*<* 0.39 at baseline are more likely to exhibit favorable post-PEGPH20 changes in DW-MRI and DCE-MRI metrics, including perfusion-related parameters.

A caveat is that we utilized volumetric RC rather than a pixelwise RC for analysis of DCE-MRI parameters, which may have resulted in overestimate of the fraction of p-responder pixels in the tumors we analyzed. That being said, in a limited repeatability analysis, tumor parameter values were highly correlated between tumor annotations independently made by two observers.

In summary, our DW-MRI and DCE-MRI studies indicate that treatment with PEGPH20 produces a release of interstitial water along with improved penetration of the tumor by intravenously administered small molecule gadolinium-based MRI contrast agent. We have identified MRI biomarkers to guide the pre-selection of patients, and for non-invasively monitoring tumor response to co-treatment with PEGPH20. Elevated tumor HA correlates with poorer prognosis in several cancers besides PDA, such as breast, gastric, colorectal, ovarian, prostate, and lung^12^. We enthusiastically look forward to the results of new and emerging studies of PEGPH20 where treatment has previously been shown to be well tolerated^24^. We believe that future clinical trials of PEGPH20 and other stromal modifiers should incorporate acquisition of quantitative MRI, and anticipate that the predictive and prognostic MRI biomarkers presented here will inform these future studies.

## Methods

### Clinical and MRI study design

We collected MRI data in three clinical trials of PEGPH20: HALO-109–101 (NCT00834704), HALO-109–102 (NCT01170897), and HALO-109–201 (NCT01453153). The studies were conducted at five centers in the United States and four centers in Russia in accordance with the Declaration of Helsinki and Good Clinical Practice Guidelines of the International Conference on Harmonization, and was approved by the local institutional review board at each study site. Written informed consent was obtained from all patients. In all studies, eligible patients were at least 18 years of age. In HALO-109–101 and HALO-109–102, eligible patients had diagnoses of pathologically confirmed, measurable, metastatic or locally advanced solid tumors refractory to standard treatment^5^. In HALO-109–201, eligible patients had a Karnofsky score of ≥70% and a life expectancy of at least 3 months with newly diagnosed, previously untreated, histologically confirmed stage IV PDA and documented metastasis to the liver and/or lung^6^.

HALO-109–101 was a Phase I dose-escalation study (0.5–50 *μ*g/kg) on 14 patients, with PEGPH20 administered once or twice weekly on day 1 (and 4) of each 21 day cycle^5^. Baseline HA staining was obtained in 7 subjects. DW-MRI was obtained in 12 subjects, 10 with imaging done both before and 2–4 days after PEGPH20.

In HALO-109–102, PEGPH20 was administered (0.5–5 *μ*g/kg) once or twice weekly on day 1 (and 4) in 25 day cycles together with the anti-inflammatory dexamethasone administered one hour before PEGPH20 in 26 subjects^5^. HA staining before and after drug (day 2 or beyond) was available in 6 patients. DW-MRI was obtained in 16 subjects, of whom 13 subjects had available DW-MRI at baseline and following PEGPH20 (day 1; days 2, 3 or 4, and end of first cycle). On the same dates, pre-contrast T1-weighted (T1w)-MRI and DCE-MRI were obtained in 16 subjects, of whom 13 subjects had imaging available at baseline and following PEGPH20.

In HALO-109–201, 28 subjects received escalating intravenous doses of PEGPH20 (1, 1.6, 3 *μ*g/kg) in combination with GEM (1,000 mg/m^2^ i.v.; Sun Pharmaceuticals) using a standard 3 + 3 dose-escalation design. In cycle 1 (8 weeks), PEGPH20 was administrated twice weekly (days 1 and 4) for 4 weeks, then once weekly for 3 weeks. GEM was administrated 24h after the first dose of PEGPH20, and all other doses were given 2 to 24 hours after PEGPH20 once weekly for 7 weeks, followed by 1 week off treatment. In each subsequent 4-week cycle, PEGPH20 and GEM were administered once weekly for 3 weeks, followed by 1 week off. Dexamethasone (8 mg) was given 1h before and 8–12h after PEGPH20 administration. Twenty patients had a baseline biopsy assessed for HA content^6^. Pre-contrast T1w-MRI and DCE-MRI series were obtained before the first dosing on day 1 of week 1 in addition to 8 and/or 24 hours after the first dose of PEGPH20 in Cycle 1, and then 24 hours after the last dose of PEGPH20 in Cycle 1 (Week 7). 7 subjects were imaged, with 6 of these subjects having imaging available at baseline and following PEGPH20. A CONSORT diagram is shown in Fig. 1 of Supplementary Material A.

### Tumor HA immunohistochemistry

On available tumor biopsies, degree of positivity for HA was semi-quantified using either a pathologist visual scoring method (H-score) or digital image analysis^5,6^. Biopsies of tumors visible on qMRI could be analyzed for correlation with their qMRI parameters (see Table 1).

### MRI acquisition

Single-shot EPI DW-MRI images were acquired with typically 20 slices reconstructed to a matrix size of 256×256, in-plane resolution of ~1.5×1.5 mm^2^, slice thickness of 8 mm, TR ~6 s, TE ~80 ms, field strength 1.5 T, FA 90°, and *b* values of 0 and 450 s/mm^2^. For the expected types of metastatic cancers, these b values represent a good compromise^25^ and were fixed to these two values in the imaging manual in order to maximize adherence to the imaging protocol in these multi-site, multi-country studies that involved primarily non-academic sites without robust in-house technical expertise. Typically, 3 replicates of the DW-MRI were obtained per session.

Pre-contrast 3D Gradient Echo (3D-GRE) T1w-MRI images were acquired with typically 12 slices reconstructed to a matrix size of 256×256, in-plane resolution of ~1.3×1.3 mm^2^, slice thickness of ~5 mm, TR ~3 ms, TE ~1 ms, field strength 1.5 T, and FA = 15°, 23°, 30°, and 60°. The DCE-MRI series comprised of *>* 40 3D-GRE T1w-MRI repeats acquired every ~8 s with the same parameters as the pre-contrast T1w images but with a fixed FA = 30°. Gadolinium contrast (0.1 mmol/kg) was power-injected at 4 mL/s after 4–10 repeats had been collected in the T1w dynamic series, and chased with 20 mL saline at 4 mL/s.

### Image post-processing

All DW-MRI images per session were co-registered, and ADC was computed per replicate using the mono-exponential model^26^. A mean ADC per session was obtained by averaging the available ADC replicates.

Pre-contrast T1w-MRI images acquired with multiple FA and the DCE-MRI series were co-registered per session^26^. T1 relaxation time maps were calculated by fitting the T1w image intensities to the GRE signal equation while enforcing spatial smoothness in T1 maps^27^. In case of noticeable intensity inhomogeneity at the tumor site, true FA in the images were estimated before getting T1 maps^28^. Also, in case of differences in intensity scaling between T1w-MRI images, the scaling factors were estimated prior to computing T1 maps^26^. Gadolinium concentration maps were calculated from the change in pixel-wise T1 relative to baseline in the DCE-MRI series. In some cases, pre-contrast T1w-MRI images were not available or had not been acquired according to the study protocol. In these cases, reference values of T1 were used for normal tissues and/or blood. iAUC maps were computed as the area-under-the-gadolinium-concentration-curve 90s after contrast injection. To describe the contrast exchange between blood and tissue we used the extended Tofts model to obtain *k*^*trans*^, *v*_*p*_, and *v*_*e*_. The Arterial Input Function (AIF) was obtained by averaging selected contrast curves in pixels in a main artery^26^. Finally, all parameter maps including ADC were co-registered across scan dates. Per-patient HTML reports that depict all slices of raw qMRI images, processed qMRI images, and computed maps of ADC, T1, iAUC, *k*^*trans*^, *v*_*p*_, and *v*_*e*_, from all scan dates of each subject, have been publicly shared (Supplementary Material B).

### Segmentation

Study images were centrally collected at Imaging Endpoints Core Lab, and tumor and reference tissues were identified by central review. Target tumors and Volumes-of-Interest (VOIs) within normal reference tissues were then manually contoured in a blinded fashion by an experienced imaging scientist (NR) on both on DW-MRI and DCE-MRI for each visit. To evaluate robustness of tumor metrics to manual annotation, a subset of five patients were also annotated in a blinded manner by an experienced radiologist (JRC).

### Statistical Analysis

MRI data were analyzed on VOI and pixelwise basis. In VOI analysis, median ADC, T1, iAUC, *k*^*trans*^, *v*_*p*_, and *v*_*e*_ values within segmented VOIs were computed at each scan date. Following Quantitative Imaging Biomarkers Alliance (QIBA) recommendations^25^, median parameter changes per visit are identified as significant changes if these are either above or below the Repeatability Coefficient (RC), where 95% of repeated measurements should be inside the − RC + RC range. From the ADC replicates at baseline per patient and tumor, we obtained the median ADC Repeatability Coefficient (*RC* = 2.77√*σ*^2^). For patients in whom multiple replicates of the DW-MRI were not available at the baseline scan date, the whole data RC was used instead. Replicate scans were not acquired for T1w-MRI or DCE-MRI; therefore, as recommended by QIBA, RC values were obtained from literature on similar data in tumors; RC = 0.27s for T1^29^, RC = 32% for iAUC, RC=−45–83% for *k*^*trans*^, RC = 0.076 for *v*_*e*_^30^, and RC = 0.0062 for *v*_*p*_^31^. Tumors with a positive or negative (depending on the parameter) significant changes in a parameter were labeled pharmacodynamic responders (P), and otherwise labeled pharmacodynamic non-responders (N). Models to predict positive (PP) or negative (PN) tumor pharmacodynamic response were evaluated by their sensitivity = (PP ∩ P)/P, specificity = (PN ∩ N)/N, and Balanced Accuracy (BA) = (sensitivity + specificity)/2. A similar assessment was performed for pixel-wise analysis of tumors from patients with available co-registered MRI sequences across scan dates.

## Figures and Tables

**Table 1:** Summary of patient data

	Patient ID	Dose level (μg/Kg)	Primary cancer diagnosis^1^	HA staining^2^	Response^3^	Lesions on DW-MRI	Lesions on T1w-MRI	Lesions on DCE-MRI

HALO-109-101	101-001-109	0.75	Ovarian	-	SD	1	0	0
	101-002-102	50	CRC	-	PD	1	0	0
	101-002-106	0.5	CRC	-	PD	1	0	0
	101-002-114	1.5	Prostate	-	PD	1	0	0
	101-003-103	0.5	CRC	-	AE	8	0	0
	101-003-104	0.5	Pancreas	10%,0,150→ -	PD	1	0	0
	101-003-105	0.5	Bladder	-	PD	3	0	0
	101-003-108	0.75	Carcinoid	-	PD	5	0	0
	101-003-110	0.75	CRC	-	PD	1	0	0
	102-003-112	1	NSCLC	-	PD	1	0	0

HALO-109-102	102-092-102	1.6	Esophageal	-	PD	1	0	1
	102-092-103	5	NSCLC	-	AE	0	1	1
	102-002-113	3	Pancreas	-	AE	1	1	1
	102-002-115	3	Pancreas	-	AE	1	1	1
	102-002-126	3	CRC	-	SD	1	1	1
	102-003-104	1.6	CRC	T2: 17.9%,-,- → 3.5%,-,-	PD	2	2	2
	102-003-105	1.6	CRC	-	PD	5	2	2
	102-003-117	3	CRC	12.2%,-,- → 20.8%,-,-	PD	1	1	1
	102-003-118	3	Tonsil	-	PD	1	1	1
	102-005-111	3	CRC	-	AE	1	1	1
	102-006-107	3	Pancreas	-	PD	1	1	1
	102-006-110	3	CRC	-	A E	1	1	1
	102-006-124	3	CRC	-	PD	1	1	1

HALO-109-201	201-901-301	3	Pancreas	-,280,270→ -	PR	0	1	1
	201-001-304	3	Pancreas	-	PR	0	1	1
	201-003-306	3	Pancreas	-	SD	0	1	1
	201-007-405	3	Pancreas	T1: -,40,260→ -,15,150	PR	0	3	5
	201-007-409	3	Pancreas	-,100,240→ -	PR	0	1	1
	201-007-414	3	Pancreas	T1: -,30,190→ -	PR	0	5	5

	# Patients: 29				# Lesions:	40	26	29

1 CRC: Colorectal Cancer, NSCLC: Non-small-cell lung carcinoma

2 Tumor biopsied (in case of multiple tumors): HA%, tumor, and stroma H-score before and after PEGPH20

3 Response by RECIST. SD: Stable Disease, PD: Progressive Disease, PR: Partial Response, AE: discontinuation of the study due to Adverse Events.

## Data Availability

Per-patient HTML reports that depict all slices of raw qMRI images, processed qMRI images, and computed maps of ADC, T1, iAUC, *k*^*trans*^, *v*_*p*_, and *v*_*e*_, from all scan dates of each subject, have been publicly shared (link in Supplementary Materials B). De-identified raw MRI images from the studies reported here are available from the corresponding author upon request and approval by Halozyme Therapeutics. MATLAB code for reproducing the image processing reported here are available from the first author or the corresponding author upon request.
